# The 2017 M Balasegaram Memorial Lecture: The Changing Landscape of Liver Diseases in Malaysia—60 Years On!

**DOI:** 10.21315/mjms2019.26.2.3

**Published:** 2019-04-30

**Authors:** Khean-Lee Goh

**Affiliations:** Department of Medicine, Faculty of Medicine, University of Malaya, Kuala Lumpur, Malaysia

**Keywords:** liver abscess, hepatitis B, hepatitis C, hepatocellular carcinoma, non-alcoholic fatty liver disease, Malaysia

## Abstract

The landscape of liver diseases in Malaysia has changed dramatically since the time of Professor Balasegaram Manickavasagar—an eminent surgeon in the 1960s.

The most significant discoveries in hepatology have been that of hepatitis B virus in 1963 and hepatitis C virus in 1989, which have both been shown to be predominantly blood borne diseases.

Hepatitis B and C infections result in long term carrier state and a high propensity to develop liver cirrhosis and cancer. Hepatitis B is the most common cause of liver cirrhosis and cancer in Malaysia. Blood bank screening and public health preventive measures have reduced the disease burden significantly and an effective vaccination for hepatitis B is now incorporated in our National Immunisation Programme. Although no vaccine is available for hepatitis C, highly effective eradication therapies were introduced in 2011. These agents will significantly change the disease scenario across the world.

A “new” disease was described in 1980, by Ludwig et al.—non-alcoholic fatty liver (NAFLD) disease. With the global epidemic of obesity and diabetes mellitus, NAFLD is set to increase exponentially across the world including in Malaysia. It will be the most important liver disease in the future, replacing hepatitis B and C infections.

## Introduction

Professor Balasegaram Manickavasagar was a legend in his own time. Not only was he a pioneer in liver surgery, he was also a pioneer in hepatology in Malaysia. My first ‘contact’ with him, was when I read his paper on the treatment of liver abscess (1).

I was writing an article, on our experience with liver abscess at the University Hospital, Kuala Lumpur, now known as University of Malaya Medical Centre (UMMC), in the mid-1980s (2). Liver abscess was a common clinical problem at that time, when I first started working as a lecturer in the University of Malaya in 1984. Diagnosis was made principally on clinical grounds and supported by findings on Technitium^99^ radionuclide scanning of the liver. Ultrasonography and CT scanning were in its infancy and not widely available in clinical practice. Liver abscesses were sometimes so advanced that there would be a ‘pointing’ sign on the skin. Senior physicians in the ward would sometimes put in a metal trocar to drain the abscess at the bedside. Many cases were treated by open surgical drainage. Most of these cases of liver abscess were thought to be amebic in origin but its etiology was seldom proven (2). With increasing affluence and modernisation, amebiasis and amebic liver abscess have now declined dramatically and have become uncommon diseases in urban Malaysia.

By the mid-1980s, Professor Balasegaram had already published widely on his experience in liver abscesses (1, 3). Dr Mark Ravitch, Editor-in-Chief of the journal *Current Problems in Surgery* wrote in his forward to Professor Balasegaram’s treatise on liver abscess (1) that “Professor Balasegaram, as his monograph shows, has extraordinary experience with abscesses of the liver, both pyogenic and amebic. In this modern age, the tools of diagnosis, whether sophisticated bacteriology, sonography, or computer assisted tomography, are as available in Kuala Lumpur as in the United States.” His peer recognition was wide and at the highest levels. He received the Hunterian Professorial Award in 1969 as well as the Jacksonian Prize in 1971 from the Royal College of Surgeons of England (4). There were numerous other accolades conferred upon him.

**Figure 1:**
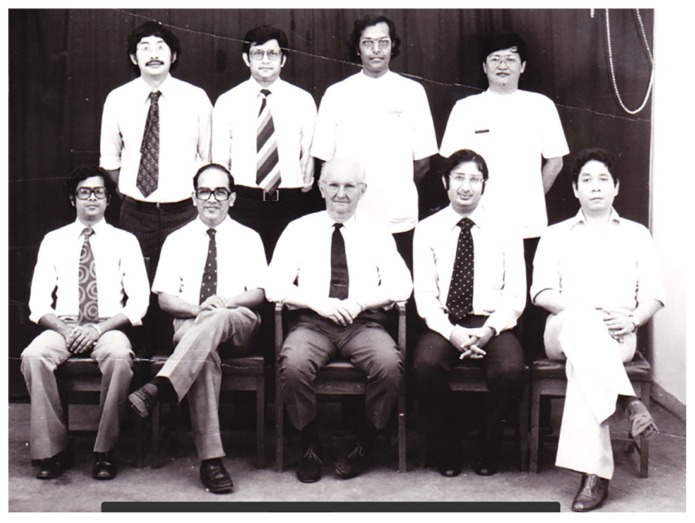
Professor M Balasegaram (front row, seated 2nd from left) and his doctors with a distinguished visitor; circa 1969; courtesy of Mangai Balasegaram

Professor Balasegaram’s expertise was not just confined to liver abscess. He was a general and liver surgeon par excellence. As Professor Dato P Kandasami, one of his pupils, always says, Professor Balasegaram had ‘magic’ or in Tamil language ‘kairasi’ in his hands. He worked in Seremban General Hospital as Chief of Surgery before moving in the late 1960s to become Head of Surgery in the General Hospital Kuala Lumpur. He trained many surgeons, many of whom had lived up to his highest expectations and have become top class surgeons in their own right (Figure 1). On the back of his tremendous clinical work, he published several seminal articles on liver surgery (4–7). He also embarked on experimental liver transplantation on dogs in the Seremban hospital in the late 1960s. He had commented then, that in three conditions “the advanced multicentric hepatomas, the failing cirrhotic livers and bilary atresia, partial hepatic resection had little to offer and only liver transplantation offered a viable treatment option” (4).

Professor Balasegaram was always ahead of his time and was an accomplished writer, with an enviable list of scientific publications. He had started publishing in 1963 and until his last published article in 1997, he had amassed a total of 66 papers.

When I started working as a lecturer in Medicine at the University of Malaya, in 1984, I had little notion that I would become deeply involved in clinical research and enjoy publishing papers as much as I have. Professors TG Loh and then Florence Wang, who were successive Heads of Medicine at that time in the University of Malaya, had inculcated upon young academic staff the importance of publishing. But I was initially, not too enthused with writing papers. I did some out of a sense of obligation and duty as a young lecturer. I was keen to be a top class therapeutic endoscopist. My life changing year, was when I took a university sabbatical in 1991. I was accepted as a therapeutic endoscopy fellow at the famous Gastroenterology and Gastrointestinal Endoscopy unit of the Academic Medical Center (AMC), University of Amsterdam, under the tutelage of the eminent Professors Guido Tytgat and Kees Huibregtse. The AMC was the ‘Mecca’ of therapeutic endoscopy and gastroenterology at that time.

Professor Huibregtse who was Chief of Endoscopy, was the finest endoscopic retrograde cholangiopancreatography (ERCP) endoscopist in the world and watching him perform cases was always ‘easy on the eye’. Performing cases ourselves was a different ‘cup of tea’. Most fellows however, with time, soon learned from the ‘Master’ and we became quite adept at performing ERCPs well! Professor Tytgat was the overall head of the Gastroenterology Department. His energy and enthusiasm for work and scholarship was thoroughly infectious. He was often quoted as saying—‘You can talk endlessly about your experience (or clinical findings) but it does not mean anything. You have to publish it, then it belongs to you forever!’ During the time of my sabbatical, Professor Anuar Zaini who was Dean of Faculty of Medicine at that time and a most sincere and noble man, had strongly encouraged me to write a doctoral thesis. Through the advice of my senior colleague and close friend, Dr Damian Wong and on the insistence of Professor Tytgat, I decided to write-up a doctoral thesis on my work in *Helicobacter pylori* infection (8). Professor Tytgat was my external supervisor to the thesis and I obtained my ‘Doctor of Medicine (MD)’ degree in 1996.

I was fortunate to have good mentors! Throughout my professional and academic life I have always tried to reciprocate this, to all my junior staff and trainees. Mentoring has brought me the greatest joy and satisfaction in my professional career and has been, perhaps the most meaningful of all my ‘duties’ in my 36 years at the University of Malaya.

## Liver Diseases in the Early 1960s

In the 1960s, our knowledge of chronic liver diseases was centred on advanced liver cirrhosis, liver failure and liver cancer. Patients presented with jaundice and other overt signs of liver disease. Apart from alcohol intake, no other major etiological agents were reliably known. Liver trauma from motor vehicle accidents and other blunt injuries were increasingly seen by the surgeons in the late 1960s.

Patients frequently died from these injuries, until timely surgical intervention managed to save many lives. Professor Balasegaram who was Head of Surgery at the General Hospital Kuala Lumpur, saw many such cases in the late 1960s and was at the forefront in treating these cases (9, 10).

Acute hepatitis was diagnosed only when jaundice was present. An infectious etiology was suspected and acute hepatitis was divided into two types—infectious hepatitis (type A, MS-1) with an incubation period of 15–45 days and a serum hepatitis (type B, MS-2) with a longer incubation period of 45–160 days (11). Type A hepatitis was also referred to as “epidemic hepatitis” and was recognised from the 17th century onwards, by its occurrence in military camps during times of war. It was thought to be spread by overcrowding and poor hygiene. Type B hepatitis was first described in the 19th century when inoculation for smallpox resulted in many cases of jaundice. In the early part of the 20th century, epidemics of jaundice occurred following injections for treatment of venereal diseases, diabetes mellitus and tuberculosis. Cases were also reported following blood transfusions and in children who received convalescent serum for prevention of mumps and measles. Contaminated syringes and needles were incriminated as the source of transmission of the virus or infective agent. In the late 1950s, Krugman and colleagues carried out ‘human experiments’ in the Willowbrook State School for mentally defective children, where there was endemic hepatitis. Although the putative ‘viral’ etiology was not confirmed, they carried out important studies on transmission of infection, on the course of disease as well as on prevention of infection with the use of gamma globulins (12, 13). Further research into the etiology of hepatitis, was however, hampered by the inability to propagate the ‘virus’ in tissue culture or in experimental animal models.

Chronic liver diseases were poorly understood. Sub-entities of chronic persistent and chronic active hepatitis were described in the textbooks denoting liver diseases of differing ‘severity’. In Sheila Sherlock’s famous and well-read textbook—*Diseases of the Liver and Bilary System*, she wrote that ‘The progression of virus hepatitis to a chronic disease is probable but is still not unanimously accepted’ (14). In our present state of knowledge of viral hepatitis, it is almost unimaginable that such a statement could have been made then. The notion of asymptomatic chronic viral hepatitis or ‘healthy carrier’ did not exist.

## Changing Landscape of Liver Disease

### Discovery of the Hepatitis B Virus

**Figure 2:**
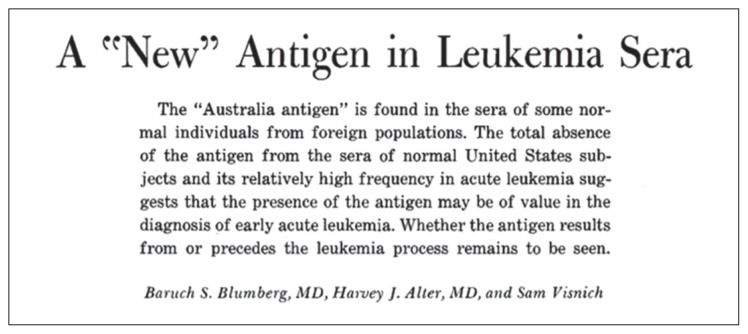
The original description of the ‘Australia antigen’ by Blumberg et al. (15)

One of the most significant discoveries in liver disease in the modern era was entirely serendipitous. Blumberg and colleagues working from the Institute of Cancer Research in Philadelphia and the National Institutes of Health in Bethesda, USA described in 1965, a high prevalence of antibodies in sera of leukemia patients cross reacting with an antigen found in the serum of an Australia aborigine (15) (Figure 2). They called it ‘the Australia antigen’. Blumberg et al. had no idea that this was the hepatitis B virus and thought that the Australia antigen was a pre-leukemia or a susceptibility marker to leukemia. A subsequent paper showed that there was also a high prevalence in patients with Down’s syndrome and ‘viral’ hepatitis as well (16). Further studies eventually showed that the Australia antigen was identical to the serum hepatitis virus (17). In 1970, Dane and colleagues visualised the outer coat of the virus as well as the whole virus particle on electron microscopy (18). Rapid developments in the laboratory followed quickly, particularly that of the solid phase sandwich radioimmunoassay, which allowed accurate and rapid detection of the hepatitis B surface antigen (19). Screening of large samples of blood in the population such as in blood banks then, became possible. Importantly, the reliable diagnosis of hepatitis B virus infection allowed us to understand clearly, the epidemiology and natural history of the infection.

### Hepatitis B Vaccine Development

Vaccination towards hepatitis B infection was first introduced in the 1980s. The first generation vaccines were all plasma derived (20). These vaccines however suffered from the risk of transmission of other blood borne viruses such as the HIV virus as well as the difficulty in producing adequate amounts for widespread use. Bioengineering techniques, using yeast cells were used to produce the major segment of the hepatitis B virus protein inexpensively and in large amounts in the early 1980s. This was a great advance in the practical implementation of a global vaccination programme (21, 22). Vaccination in Malaysia was given to all newborns since 1989 when it has subsequently been incorporated into the National Immunisation Programme. The WHO recommended universal childhood vaccination for hepatitis B, globally in 1992 (23). The introduction of hepatitis B vaccination by reducing the huge reservoir of the infection, was a major step in the control of the disease.

### Epidemiology and Natural History of Hepatitis B Infection

**Figure 3:**
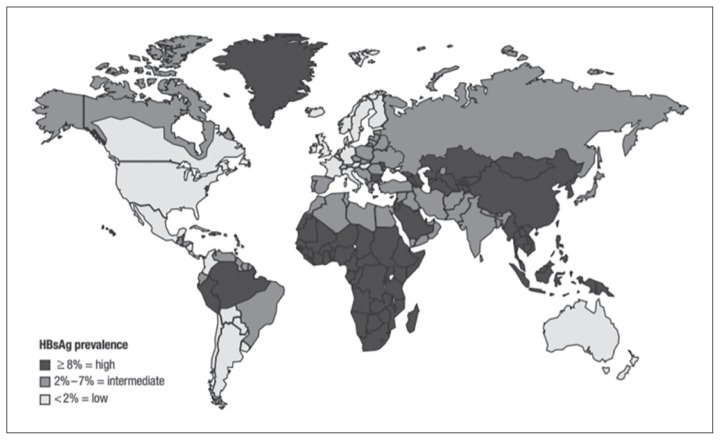
Global distribution of hepatitis B virus infection (25)

Hepatitis B infection is one of the most prevalent infection globally, with the highest burden in East Asia and in Africa. Worldwide, an estimated 257 million of the world’s population harbour the virus (24). The highest prevalence is found in the Asia-Pacific region, Africa and in South America (25) (Figure 3). Hepatitis B infection is transmitted through blood transfusions of contaminated blood as well as direct inoculation intravenously or into tissues. It also became apparent that that the epidemiology is different in Asian compared to Caucasian patients. In Asian patients, infection usually occurred during the neonatal period (26). It was therefore confined to families and transmission was within families.

Neonatally acquired infection results in long term carrier state (27). In contrast, amongst Western patients, infection takes place in adolescence and adulthood. The disease is of short duration and seldom results in a carrier state (28, 29).

Long term carriage of hepatitis B virus, results in the development of cirrhosis and hepatocellular carcinoma in a significant proportion of patients (30). The role of hepatitis B viral infection in the causation of liver cancer has been clarified over the years. Many of these seminal studies, were carried out in Taiwan where hepatitis B infection is highly prevalent. Beasley and colleagues studied a large cohort of over 20,000 government employees in Taiwan and reported their results in 1981 (31). This study followed-up subjects prospectively for the development of hepatocellular carcinoma and calculated a phenomenal increased risk of > 200 fold in hepatitis B carriers compared to non-carriers. A plethora of studies have followed, including seminal basic laboratory studies which have shown integration of HBV DNA sequences into cellular DNA of human hepatocyte genome (32). Another seminal study from Taiwan, reported on the effect of mass vaccination of children with the hepatitis B vaccine on childhood liver cancer (33). This study showed a dramatic decline in the incidence of childhood liver cancer within a period of 10 years. A later study confirmed the efficacy of this programme in decreasing hepatocellular carcinoma in the whole Taiwanese population (34).

Hepatitis B virus infection is now established as a major cause of liver cancer globally and particularly in the Asia-Pacific region. Our own studies, have shown that hepatitis B infection is the major cause of cirrhosis of the liver and hepatocellular carcinoma in Malaysia (35, 36).

### Hepatitis A Virus

The discovery of the hepatitis A virus was comparatively easier. Previous attempts to find the virus in the serum of patients had been unsuccessful as the duration of infection was short and the viral burden, low in magnitude. In contrast, studies had shown that faeces of patients were infective for 2 weeks before and after the onset of infection. In 1972, Stephen Feinstone visualised the virus particles in the stools of patients who were suffering from infective hepatitis (37). Shortly after the discovery, a serological diagnostic test became available. The epidemiology of hepatitis A, fits in with the classical infective hepatitis with a fecal-oral route of spread with many patients developing clinically apparent acute hepatitis. No carrier state is reported with hepatitis A infection.

### Transfusion Associated Non-A Non-B Hepatitis

Hepatitis B virus was originally deemed the ‘serum hepatitis’ virus being the putative agent for blood related transfusion hepatitis. However, the prevalence of transfusion associated hepatitis only decreased by 25%–50%, following exclusion of hepatitis B blood from the blood donor pool (38, 39). A third infectious agent, aptly called, the non-A, non-B hepatitis (following the discovery of hepatitis A and B viruses and their exclusion in the diagnosis), was suspected. The search for this agent, using multiple laboratory techniques and for many years, met with little success. Non-A, non-B hepatitis was however, by then, a well-recognised clinical entity with a predominantly subclinical infection and a high proportion of serious chronic sequelae (40).

### Discovery of the Hepatitis C Virus

**Figure 4:**
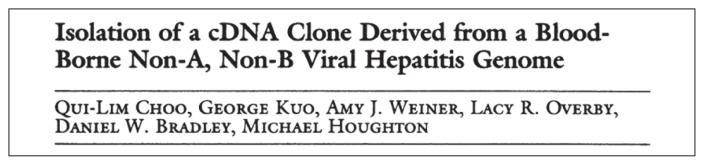
Report on the discovery of the hepatitis C virus (41)

The discovery of the hepatitis C virus in 1989 was a landmark breakthrough in hepatitis research. Using novel molecular biology techniques and animal transmission studies, Choo and Houghton working from the Chiron Corporation, California, USA and in collaboration with the Center for Disease Control (CDC), were able to identify a genetic sequence (clone 5-1-1), following screening of hundreds of millions of plasma and liver samples of infected chimpanzees which cross reacted with antibodies from a human non-A, non-B hepatitis patient (41) (Figure 4). This genetic sequence was identified as part of the hepatitis C virus. Together with this discovery, an antibody test was developed at the same time (42).

Diagnostic serological testing for antibodies to hepatitis C became commercially available soon after, in 1991.

### Epidemiology of Hepatitis C Infection

**Figure 5:**
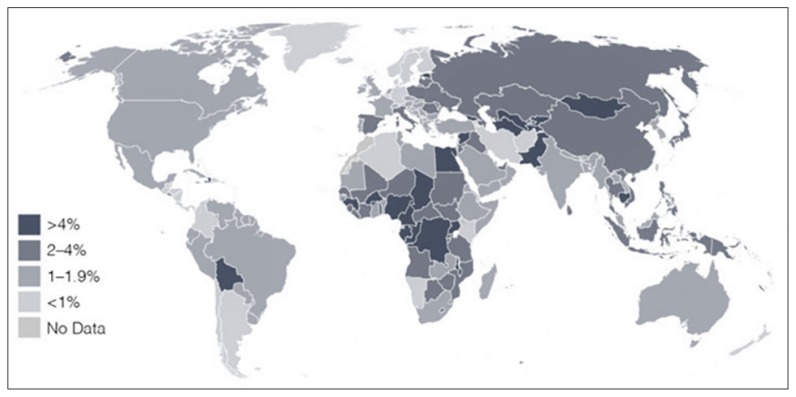
Global distribution of hepatitis C virus infection (44)

Widespread testing, demonstrated the global epidemiology of this infection. Hepatitis C is a subclinical infection leading to chronicity in a majority of patients. Approximately 71 million of the world’s population is thought to be infected presently (43). Hepatitis C infection is seen mainly in the poorer and underdeveloped countries in the world (44) (Figure 5). It is the most common cause of chronic liver disease in the Western world and in Japan and the hepatitis C related diseases, the most common indication for liver transplantation.

Although a vaccine has not been developed because of the existence of a large number of quasi species of the virus, treatment of the infection had been gratifying. Immune modulating agents, alpha interferon in combination an antiviral drug, ribavirin, were originally used for more than decade, with modest eradication rates. In 2011, the introduction of new direct antiviral agents (DAAs) has been a true ‘revolution’ in the treatment of hepatitis C (45). The use of combination of these new drugs, has been a phenomenal success with eradication rates of close to 100%. With greater affordability and widespread use of these drugs, total elimination of the virus is now, a distinct possibility.

### A New Disease—Non-Alcoholic Fatty Liver Disease

In 1980, Ludwig and colleagues described a ‘new disease’ and called it ‘non-alcoholic steatohepatits’. They wrote in their paper, that it was ‘a poorly understood and hitherto unnamed liver disease that mimics alcoholic hepatitis that also may progress on to cirrhosis’ (46). Non-alcoholic fatty liver disease (NAFLD) is the more generally used term and can now be broadly divided into benign steatosis and non-alcoholic steatohepatitis. NAFLD is predicted to become the most common liver disease globally, in the near future (47). In the Asia-Pacific region, an epidemic of diabetes mellitus and overweight/obesity marks NAFLD as a very important disease affecting large segments of the population (48). NAFLD is a common disease in Malaysia affecting particularly a high proportion of Malays and Indians in the population (49, 50). The natural history of NAFLD has been well described and its progression to cirrhosis and liver cancer is well documented (51).

## What of the Future for Liver Diseases?

The clear understanding of the transmission of infection of viral hepatitis B and C through a predominantly parenteral route, has resulted in the implementation of effective public health preventive measures such as safe inoculation and disposal of needles and sharps. The global burden of deaths from viral hepatitis has been estimated to have increased from 0.89 million in 1990 to 1.35 million in 2013 (52). The World Health Organization (WHO) has taken the lead in an active educational and advocacy campaign to eliminate both viral infections (53).

For hepatitis B infection, mass vaccination has already been introduced in many countries across the world, for many years. In most countries, it has become part of their ‘Expanded Programme on Immunisation’ (EPI) programme. This has markedly shrunken the reservoir of infection. Although hepatitis B related chronic disease will persist beyond 2040, the numbers will have also declined to a very low level by that time. In the meantime, antiviral treatment has been available for treatment of hepatitis B infection since 1998. Although these agents are effective in reducing the viral load, it is essentially a suppressive therapy which does not eradicate the virus. Antiviral treatment has reduced the number of patients with cirrhosis and cancer of the liver related to chronic hepatitis B infection (54, 55). Much research is going on currently to look for agents that can eradicate the virus (56).

The dramatic revolution in treatment from the introduction of DAAs in 2011, has also resulted in a sharp decline in the numbers of chronic hepatitis C patients from 170 million to less than 100 million in 5 years. Driven by local government initiatives as well as efforts of bodies such as the Drugs for Neglected Diseases Initiative (DNDi), highly effective eradication treatment for hepatitis C will soon become available across the world.

But without question, NAFLD will be the disease of the future. Fatty liver is a disease that is closely linked to obesity which is now formally recognised as a disease by the WHO.

## Conclusion

### Hepatology in Malaysia

When I started working in gastroenterology in 1984, liver disease was only a small part of our clinical practice. At the University Hospital, Kuala Lumpur, when screening for hepatitis B was implemented in 1983, a small clinic on a Saturday morning was started to review all hepatitis B surface antigen positive blood donors. We kept these patients on regular follow-up in the clinic. Until 2000, we had no effective treatment for hepatitis B. The introduction of effective antiviral drugs, albeit a suppressive therapy, has changed the outcome of patients with chronic hepatitis B virus infection significantly. Hepatitis B vaccination at birth for all children, was implemented nationwide in 1989 and is now incorporated in the National Immunisation Programme.

Testing and treating hepatitis C patients have entered a new phase with the introduction of ‘compulsory licensing’ for DAAs in Malaysia in 2018. Many patients would now be able to receive these highly effective treatment at an affordable price.

The heavy burden of chronic liver diseases due to hepatitis B and C will continue for at least for the next 20–30 years. The population who were not protected by hepatitis B vaccination before 1989 will be only 60 years of age 2050. Hepatitis C eradication treatment is being implemented now and elimination of the pool of carriers will take perhaps 10 years or more. Many patients have already carried the virus for many years and a significant proportion will develop cirrhosis and liver cancer in the years to come.

Tan Sri Dr Mohd Ismail Merican, the Director-General of Health, Malaysia at that time, was instrumental in pushing the agenda for liver diseases in Malaysia in a major way. He started the Malaysian Liver Foundation in 1997 and a dedicated liver unit at the Selayang Hospital, the first of its kind in Malaysia, in 1999.

‘Surgical’ liver diseases were dealt with rather more effectively from the beginning. Professor Balasegaram was already successfully resecting tumors in the early years. With the advent of better radiological imaging of the liver, the approach to surgical treatment of liver tumors have improved.

Liver abscesses for example, do not undergo surgical resection nor drainage anymore but through imaging guided percutaneous catheter drainage. The field of interventional radiology started to grow rapidly, with technological advances in ultrasonography, CT and MRI scanning. Interventional radiologists have become involved in treatment of liver cancers with sophisticated procedures such as radiofrequency ablation and trans-arterial chemoembolisation.

**Figure 6:**
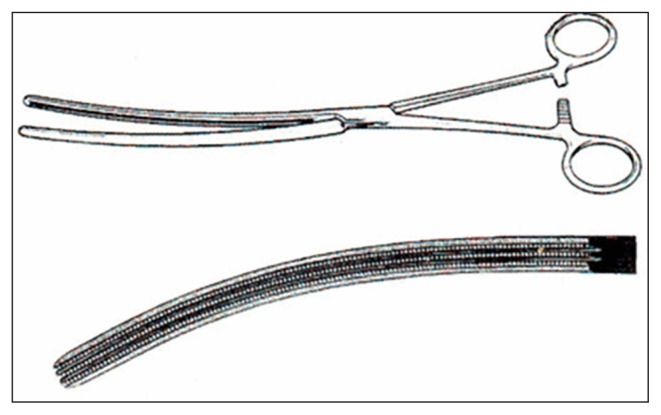
Diagram of the Balasegaram liverclamp Courtesy of Mangai Balasegaram

Liver surgery has become a specialised field as more cases are carried out. Surgeons have become more skilled, following on advances such as the Bala clamp, introduced almost 60 years ago (Figure 6). However, the ultimate treatment in liver disease—liver transplantation was slow to come on in Malaysia. Liver transplantation was first performed by Thoma Starzl in 1963. Thirty years after Professor Balasegaram’s experiments with liver transplantation in dogs, in the year 2002, Dr Harjit Singh and Dr R Krishnan pioneered liver transplant in Malaysia at the Selayang Hospital. In 2015, Dr BK Yoong and his team performed the first living donor-related liver transplantation in an adult patient, at the UMMC.
